# Development and verification of a combined immune- and cancer-associated fibroblast related prognostic signature for colon adenocarcinoma

**DOI:** 10.3389/fimmu.2024.1291938

**Published:** 2024-01-19

**Authors:** Jingsun Wei, Xiaoxu Ge, Yucheng Qian, Kai Jiang, Xin Chen, Wei Lu, Hang Yang, Dongliang Fu, Yimin Fang, Xinyi Zhou, Qian Xiao, Yang Tang, Kefeng Ding

**Affiliations:** ^1^Department of Colorectal Surgery and Oncology (Key Laboratory of Cancer Prevention and Intervention, China National Ministry of Education, Key Laboratory of Molecular Biology in Medical Sciences), The Second Affiliated Hospital, Zhejiang University School of Medicine, Hangzhou, Zhejiang, China; ^2^Department of Colorectal Surgery and Oncology, Zhejiang Provincial Clinical Research Center for Cancer, Hangzhou, Zhejiang, China; ^3^Department of Colorectal Surgery and Oncology, Cancer Center of Zhejiang University, Hangzhou, Zhejiang, China

**Keywords:** immune, CAF, prognosis, colon adenocarcinoma, TME

## Abstract

**Introduction:**

To better understand the role of immune escape and cancer-associated fibroblasts (CAFs) in colon adenocarcinoma (COAD), an integrative analysis of the tumor microenvironment was performed using a set of 12 immune- and CAF-related genes (ICRGs).

**Methods:**

Univariate and least absolute shrinkage and selection operator (LASSO) Cox regression analyses were used to establish a prognostic signature based on the expression of these 12 genes (*S1PR5*, *AEN*, *IL20RB*, *FGF9*, *OSBPL1A*, *HSF4*, *PCAT6*, *FABP4*, *KIF15*, *ZNF792*, *CD1B* and *GLP2R*). This signature was validated in both internal and external cohorts and was found to have a higher C-index than previous COAD signatures, confirming its robustness and reliability. To make use of this signature in clinical settings, a nomogram incorporating ICRG signatures and key clinical parameters, such as age and T stage, was developed. Finally, the role of S1PR5 in the immune response of COAD was validated through in vitro cytotoxicity experiments.

**Results:**

The developed nomogram exhibited slightly improved predictive accuracy compared to the ICRG signature alone, as indicated by the areas under the receiver operating characteristic curves (AUC, nomogram:0.838; ICRGs:0.807). The study also evaluated the relationships between risk scores (RS) based on the expression of the ICRGs and other key immunotherapy variables, including immune checkpoint expression, immunophenoscore (IPS), and microsatellite instability (MSI). Integration of these variables led to more precise prediction of treatment efficacy, enabling personalized immunotherapy for COAD patients. Knocking down S1PR5 can enhance the efficacy of PD-1 monoclonal antibody, promoting the cytotoxicity of T cells against HCT116 cells ((p<0.05).

**Discussion:**

These findings indicate that the ICRG signature may be a valuable tool for predicting prognostic risk, evaluating the efficacy of immunotherapy, and tailoring personalized treatment options for patients with COAD.

## Introduction

Colorectal cancer (CRC) is one of the most common malignant tumors worldwide, with China and the United States ranking second and fourth, respectively, in incidence and fifth and second, respectively, in mortality (1). In 2020, it was estimated that over 1.9 million patients would be newly diagnosed with CRC, including anal cancer, resulting in approximately 935,000 deaths, accounting for roughly 10% of both newly diagnosed cancers and cancer deaths worldwide (2). CRC is mainly treated with surgery, radiation and chemotherapy, although immune checkpoint inhibitors have played an increasingly important role in its recent treatment. The KEYNOTE-177 clinical study indicated that pembrolizumab should be the standard first-line treatment for patients with microsatellite instability-high or mismatch repair-deficient (MSI-H/dMMR) metastatic CRC (mCRC) (3). Only 13% of CRC patients, however, are MSI-H, with the remaining CRC patients being insensitive to immunotherapy (4). These differences in treatment outcomes may be attributed primarily to the heterogeneity and complexity within the tumor microenvironment (TME) (5). A prognostic signature specific to the TME of CRC patients may therefore aid in the effective delivery of immunotherapy.

The TME consists mainly of blood vessels, cancer-associated fibroblasts (CAFs), the extracellular matrix (ECM), and tumor-infiltrating immune cells (6). CAFs in the TME have several critical functions, including remodeling of the extracellular matrix (ECM), engaging in reciprocal signaling interactions with cancer cells and communicating with infiltrating leukocytes (7). Both the CAFs and tumor-infiltrating immune cells in the TME are indispensable in regulating the occurrence and development of tumors. CAFs can secrete a variety of cytokines and regulate immune cells through a variety of pathways. Signals from other cells within the TME can also influence CAF function. For example, activation of T cells can induce their production of interferon-gamma (IFNγ), a cytokine that can stimulate CAFs to increase the expression of programmed death-ligand 1 (PD-L1), with PD-L1 subsequently inhibiting the activity of T cells (8).

Prognostic predictive signatures based solely on immune-related genes have been developed. For example, a prognostic signature based on immune-related genes was found to predict survival in CRC patients and may reflect the state of the TME (9). In addition, a prognostic signature was designed based on subsets of CAFs in CRC and their interactions with nonspecific immune cells (10). These findings indicate the importance of investigating the prognostic implications of interactions between the immune system and CAFs.

The present study utilized RNA sequencing to assess differential gene expression of CAFs stimulated with activated peripheral blood mononuclear cells (aPBMCs) in patients with colorectal adenocarcinoma (COAD). Immune- and CAF-related gene signatures in COAD were subjected to systematic and comprehensive integrative analyses, with the prognostic value of these signatures were analyzed. A prognostic nomogram was developed to provide a quantitative analytic tool for predicting prognostic risk in patients with COAD.

## Materials and methods

### Data acquisition

Gene expression levels and clinical information of 476 patients with COAD patients and 41 normal individuals were obtained from the Cancer Genome Atlas (TCGA) database (https://portal.gdc.cancer.gov/). In addition, gene expression levels and clinical information of 566 patients with COAD were obtained from the GSE39582 dataset in the Gene Expression Omnibus (GEO) database (https://www.ncbi.nlm.nih.gov/geo/). The latter patients were randomly allocated into two groups, a training group (70%) and a testing group (30%). The testing group in the TCGA-COAD cohort and the GEO cohort were used as internal validation sets. Immune-related genes were obtained from the ImmPort database (https://www.immport.org) (11). CAFs were stimulated by aPBMCs, and changes in expression of CAF genes were determined by RNA sequencing.

### Preparation of primary cancer-associated fibroblasts

CRC tumor tissue samples were collected from three patients of the Second Affiliated Hospital of Zhejiang University, School of Medicine. CRC tissue samples were obtained from fresh, surgically resected samples and transferred to the laboratory in phosphate-buffered saline (PBS; Gibco, Carlsbad, CA) containing 10% povidone iodine within 30 min. The tissue samples were rinsed three times in PBS containing 500 U/mL streptomycin and penicillin, minced with surgical scissors into 2–4 mm^3^ pieces and plated in 60 mm-culture plates in RPMI 1640 containing 10% fetal bovine serum (FBS; Gibco, Brazil), 100 U/mL streptomycin and penicillin and 2.5 μg/mL amphotericin B. To ensure adherence to the culture plate, the tissue specimens were not submerged in culture medium. The tissue samples were cultured at 37°C in an atmosphere containing 5% CO2, with the culture medium changed every 3–4 days. One to three weeks after plating, the proliferating fibroblasts could be observed near the minced tissue. The primary CAFs were subsequently passaged and the remaining tissues were discarded. The study protocol was approved by the ethical review board of our institution (Approval number 2022-1130), and all patients provided written informed consent for tumor resection.

### Preparation of peripheral blood mononuclear cells

Withdraw 6 ml of peripheral blood from one healthy individual, placed it in an anticoagulant tube with Ethylene Diamine Tetraacetic Acid (EDTA), and gently mixed by rocking it back and forth to prevent blood coagulation. The blood samples were collected from the Second Affiliated Hospital of Zhejiang University, School of Medicine. Fresh anticoagulant-treated blood samples were diluted 1:1 with PBS, with each sample layered onto 3 mL of Ficoll-Paque plus solution (Sigma). After centrifugation at 400 g for 15 min, the lymphocyte layer was collected and washed in PBS. Erythrocytes were eliminated with red blood cell lysis buffer, and the cells were again washed in PBS. The samples were centrifuged, and the pellets, consisting of peripheral blood mononuclear cells (PBMCs), were resuspended in RPMI1640 supplemented with 10% FBS and 1% penicillin/streptomycin and incubated overnight in a Petri dish to allow monocyte adherence. The following day, the cells in suspension were transferred to a second culture bottle. PBMCs were activated by incubation with anti-CD3/anti-CD28 dynabeads (Thermofisher, US) for 24 hours, yielding preparations of aPBMCs.

### Co-culture of CAFs and aPBMCs

PBMCs in DMEM were prepared as described above. Following centrifugation and resuspension, a 20 µl aliquot was transferred to cell counting plate (Counter Star Company) and counted with a cell counter (Counter Star Company). The PBMCs were diluted to a concentration of 150,000 cells/100 µl, with a 100 µl aliquot of diluted PBMCs transferred to each well of a 96-well plate. A suitable volume of anti-CD3/anti-CD28 Dynabeads (Gibco, Human) was washed with a magnet stand, followed by removal of the supernatant, resuspension in DMEM culture medium, and addition of a 3 µl aliquot of suspended Dynabeads to each well of the 96-well plate containing PBMCs. The experimental group co-cultured CAFs and aPBMCs in a 6-well plate for 24 hours. 2 x 10^5^ aPBMCs were placed in the upper transwell chamber (6-well plate chamber, 0.4μm pore size), with 2 x 10^5^ CAFs in the lower chamber. The control group co-cultured unactivated PBMCs with CAFs for 24 hours. The experiment was conducted with 3 biological replicates.Subsequently, samples from both groups of CAFs were collected for RNA sequencing to detect the expression level of differential genes. And the P-value<0.05 and |log2 (fold change) > 1| were considered as CAF-related differentially expressed genes (CRGs).

### Clustering of non-negative matrix factorization

Non-negative matrix factorization (NMF) is a matrix factorization technique used to divide a matrix into two non-negative matrices. DEGs in tumor and normal samples were screened out based on a |log2 (fold change) > 1| and a false discovery rate (FDR) < 0.05. DEGs correlating with prognosis were screened out by univariate COX regression analysis, and the COAD samples were classified based on the expression of prognostic relevant genes using the “NMF” package. The number of clusters K was set in the range of 2 to 10.

### Development of the combined immune- and CAF related prognostic signature

Prognostic genes in the TCGA training cohort were identified by univariate Cox regression analysis, followed by least absolute shrinkage and selection operator (LASSO) Cox regression analysis using the “glmnet” package. Based on the median risk score (RS), the training cohort was divided into two groups, a low-risk and a high-risk group. The results obtained from the TCGA training cohort were subsequently validated in the TCGA test cohort and the GEO cohort. After the construction of the ICRG prognostic signature, the resulting RS was combined with the clinicopathological information obtained from patient records, and a prognostic nomogram predicting outcomes in patients with COAD was constructed. The predictive ability of the nomogram was assessed by determining survival risks. The calibration curves were drawn using the “rms” package.

### Evaluation of the responses to immunotherapy

Comprehensive immunogenomic data were obtained from the Cancer Immunome Database (TCIA) (https://tcia.at/home). The relationships between ICRG signatures and predicted responses to treatment were analyzed based on four immune checkpoints: PD1, PD-L1,PD-L2 and CTLA4.In addition, the association between ICRG signatures and MSI was assessed to determine the efficacy of immunotherapy.

### T cell cytotoxicity assay

The plates were incubated for 48 hours to obtain aPBMCs, the anti-CD3/anti-CD28 Dynabeads were removed magnetically, and the cells were resuspended in DMEM containing IL-2 (10 ng/ml) for another 5 days. This process can cool aPBMC to prevent non-specific killing. HCT116 cells were transfected with negative control short interfering RNA (si-NC) or si-S1PR5 for 48 hours and plated at 10,000 cells per well in 96-well plates. The siRNA sequences are as follows (5’→3’): si-NC: UUCUCCGAACGUGUCACGUTT;si-S1PR5-1:CCGCUAUCUGUGCACUCUA(dT)(dT); si-S1PR5-2: CAUCGUGCUAGAGAAUCUA(dT)(dT).The next day, 40,000 aPBMCs and anti-PD1 monoclonal antibody (4 μg/ml) were added as appropriate to each well. Killed HCT116 cells were measured after 48-72 hours, with the results verified by microscopy and crystal violet staining.

### ELISA assay for IFNγ expression

After co-culturing PBMCs with HCT116, the supernatant was collected and centrifuged to remove the cells. The Human IFNγ ELISA kit (Code: EK0373, Boster, China) was used to detect the expression of IFNγ in the supernatant. The supernatant was added to each well of the enzyme-labeled plate in 100ul aliquots. The plate was then covered with a sealing membrane and incubated at 37°C for 90 minutes. After the liquid was removed from the enzyme-labeled plate, the working solution of biotinylated anti-human IFNG antibody (excluding the TMB blank colorimetric wells) was added, and the plate was sealed for another 90 minutes at 37°C. Following a wash, 100ul of ABC working solution was added to each well (excluding the TMB blank colorimetric wells), and the plate was sealed for 30 minutes at 37°C. After the wash, 90ul of TMB color development solution was added to each well and incubated at 37°C in the dark for 25 minutes. Subsequently, 100ul of stop solution was added to each well. The OD value at 450nm was measured using an enzyme immunoassay analyzer, with the TMB blank colorimetric well set as the control.

### Statistical analysis

Univariate and multivariate Cox hazard regression analyses were performed using the “survival” package of R software. Pearson correlation analysis was performed using the “corrplot” package of R software. Differences between two groups were evaluated using the Wilcoxon test, and receiver operator characteristic (ROC) curves and areas under the curve (AUC) analyzed using the “timeROC” package in R sofware. Survival outcomes were determined by the Kaplan-Meier method and compared by log-rank tests. All statistical analyses were performed using R software (version 4.2.1), with P-values <0.05 considered statistically significant.

## Results

### Classification of COAD subtypes according to the NMF algorithm

**Figure 1:**
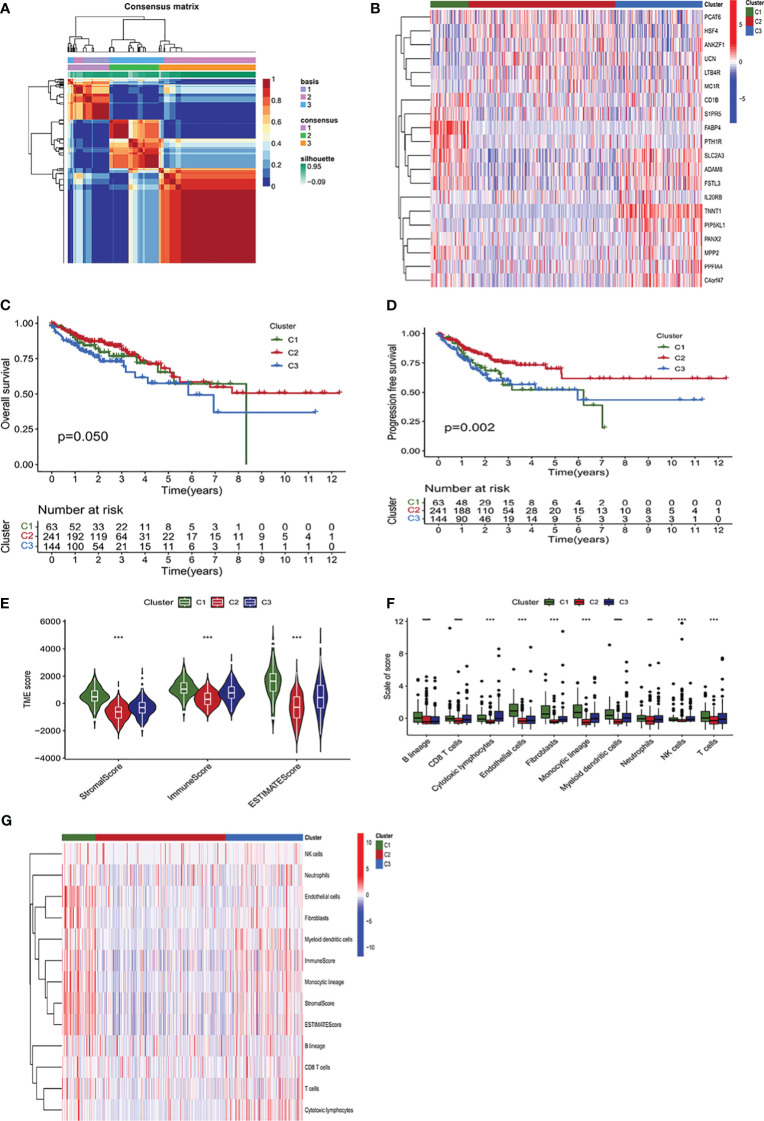
Identification of molecular subtypes of colon adenocarcinoma (COAD) using a non-negative matrix factorization (NMF) algorithm. **(A)** Heatmap of an NMF consensus matrix of K = 3. **(B)** Unsupervised clustering of the immune- and CAF-related genes (ICRGs) expression profiles of the three clusters. The heatmap displayed the expression levels of ICRG within the three clusters based on NMF classification. **(C, D)** Analysis of the differences in survival among the three clusters based on the NMF algorithm. Kaplan–Meier analysis of the **(C)** overall survival (OS) and **(D)** progression-free survival (PFS) of patients with the three subtypes of COAD. **(E)** Comparison of the TME scores of the three subtypes using the estimate algorithm. The TME scores were divided into stromalscore, immunescore, and estimatescore, with cluster 2 having the lowest scores, showing significant discrepancies (P<0.001). **(F)** Comparison of MCP counter algorithm-derived immune scores of the three subtypes. The bar chart showed the infiltration levels of immune cells in the tumor immune microenvironment of three subgroups. **(G)** Immune scores of immune cells for ESTIMATE and MCP counter algorithms displayed on the heatmap. P < 0.05, **P < 0.01, ***P <0.001, ****P<0.0001.

CAFs and aPBMCs were co-cultured for 24 hours, and gene expression levels in CAFs were measured by RNA-sequence analysis. A total of 2013 CRGs were identified, and 2483 immune-related genes (IRGs) were obtained from https://www.immport.org/ (**Supplementary Table 1**). These CRGs and IRGs were combined, with 3415 ICRGs screened during follow-up. Analysis of the levels of expression of these ICRGs in normal and colon cancer samples from the TCGA database, resulted in the selection of 1095 significantly DEGs with FDR<0.05 and |log2 (fold change) > 1|. The NFM algorithm was applied to these 1095 DEGs to identify three molecular subtypes (**Figure 1A**). The appropriate rank values were determined by analyzing the cophenetic, silhouette, and dispersion metrics (**Supplementary Figures 1, 2**), with a heatmap showing the expression of genes in the different clusters (**Figure 1B**). Kaplan–Meier analysis showed that overall survival (OS) (P=0.05) and progression free survival (PFS) (P=0.002) were significantly lower in Cluster 2 than in Clusters 1 and 3 (**Figures 1C, D**). Evaluation of the status of the TME showed that immune cell infiltration and stromal infiltration were significantly higher in Cluster 1 than in Clusters 2 and 3 (P<0.001) (**Figure 1E**). Furthermore, analysis of the infiltration of 10 types of immune cells showed that immune cell infiltration was highest in Cluster 1 and lowest in Cluster 2 (**Figure 1F**). The higher level of immune cell infiltration in Cluster 1 may indicate that this cluster was associated with a stronger immune response than the other clusters. A heatmap showed that the infiltration of endothelial cells, fibroblasts, myeloid dendritic cells and cells of the monocytic lineage was higher in Cluster 1 than in the other two clusters (**Figure 1G**).

### Construction of an ICRG prognostic signature by LASSO Cox regression analysis

**Figure 2:**
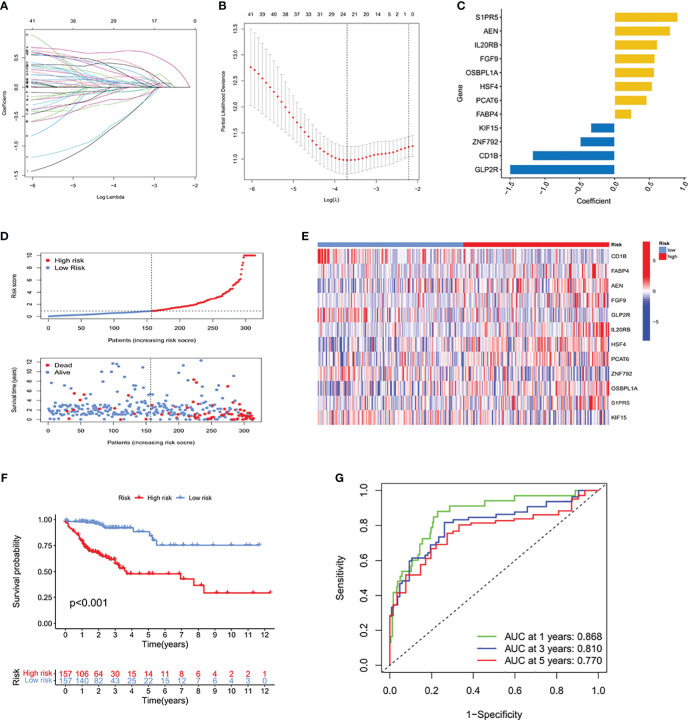
Determination of a prognostic signature for ICRGs by LASSO Cox regression analysis of the TCGA training cohort. **(A)** Determination of the coefficients of independent variables by LASSO Cox regression analysis. **(B)** Calculation of the optimal lambda value, as indicated by the first black dotted line from the left on the logarithmic scale. **(C)** Bar chart showing the correlation coefficients of each gene that constituted the ICRG prediction signature. **(D)** Distribution of risk score (RS) and survival status according to the ICRG prediction signature. **(E)** Heat map depicting the gene expression profiles of the ICRGs included in high-risk and low-risk groups based on the prognostic signature. **(F)** Kaplan-Meier analysis comparing survival rates in the high-risk and low-risk groups, which were classified based on the median RS. The prognosis of patients in the high-risk group was significantly lower than that of the low-risk group, with statistical significance (P<0.001). **(G)** ROC curves showing the predictive accuracy of the ICRG prognostic signature at 1, 3, and 5 years.

The TCGA-COAD cohort was randomly split into two subgroups, a training cohort (70%) and a testing cohort (30%), which showed no significant differences in clinical characteristics (**Supplementary Table 2**). Based on the above results, we performed univariate analysis on 1905 significantly different ICRGs and selected 47 prognostic-related ICRGs, followed by application of the LASSO-Cox regression algorithm to the selected ICRGs in the TCGA training cohort. Based on coefficients of independent variables and optimal log values of lambda in LASSO regression analysis, 23 genes were identified (**Figures 2A, B**). Risk scores (RS) were subsequently calculated by multivariate Cox regression analysis, resulting in an ICRG signature based on 12 genes (*SIPR5, AEN, IL20RB, FGF9, OSBPL1A, HSF4, PCAT6, FABP4, KIF15, ZNF792, CD1B* and *GLP2R*) (**Figure 2C**), along with their corresponding coefficients (**Supplementary Table 3**).

Based on the median RS, the TCGA training cohort was divided into two groups, those with high RS and low RS, to predict the prognosis of patients with COAD. A risk plot was generated to show played the distribution of RSs and their relationship to survival outcomes and a heatmap showed the levels of expression levels of risk genes in the high and low RS groups (**Figures 2D, E**, **Supplementary Table 4**). Kaplan–Meier analysis showed that patient prognosis was significantly lower in the high than in the low RS group (P<0.001) (**Figure 2F**). Analysis of the areas under the curve (AUCs) of the ICRG risk model showed that the 1-, 3, and 5-year AUCs were 0.868, 0.810 and 0.770, respectively (**Figure 2G**). These results showed that this prognostic model based on ICRGs had good predictive performance in patients with COAD.

### Validation of the ICRG prognostic signature

**Figure 3:**
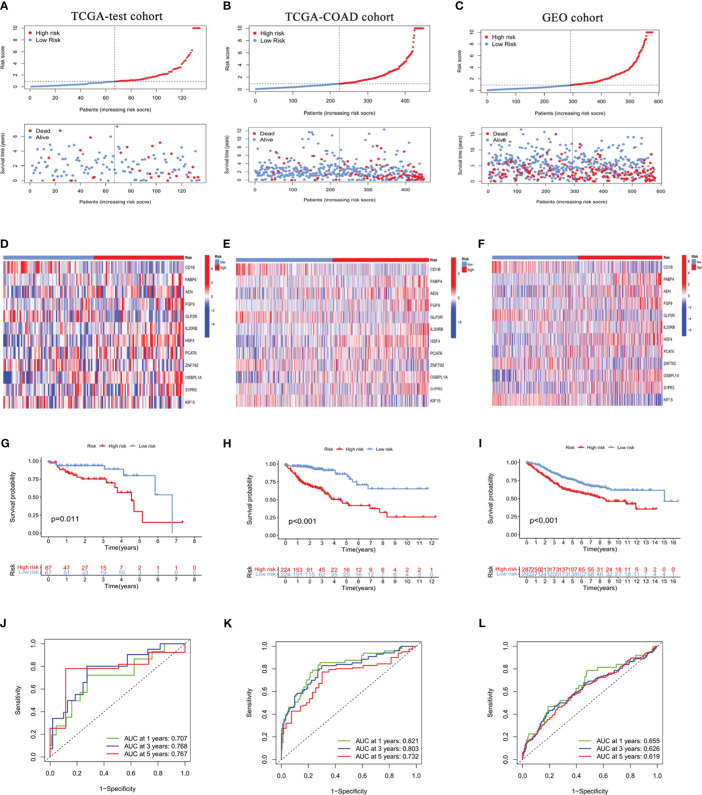
Internal and external validation of the prognostic value of the ICRG signature in the TCGA testing cohort **(A, D, G, J)**, the entire TCGA-COAD cohort **(B, E, H, K)**, and the GEO cohort **(C, F, I, L)**. **(A-C)** Distribution of risk scores (RS) and survival status in the internal and external cohorts. **(D-F)** Heat maps showing the gene expression profiles of the ICRGs in high-risk and low-risk groups. **(G-I)** Kaplan-Meier analysis comparing the survival rates in the high-risk and low-risk groups based on ICRG signature. **(J-L)** ROC curves showing the predictive accuracy of the IMRG prognostic signature at 1, 3, and 5 years.

To further evaluate the predictive value of this ICRG risk model, it was used to analyze the TCGA testing cohort and TCGA-COAD cohort for internal validation and the GEO cohort for external validation. Each of these cohorts was divided into two groups, those with high and low RS. The relationships between the distribution of risk groups and patient survival status are shown in **Figures 3A–C**, with a heatmap showing the expression of ICRGs in this risk model (**Figures 3D–F**). Kaplan-Meier analysis of survival in the testing cohorts was also performed to validate the prognostic value of this ICGR risk model. Patient prognoses were significantly higher in the low than in the high RS groups in the TCGA testing cohort (P=0.011), the TCGA-COAD cohort (P<0.001) and the GEO cohort (P<0.001) (**Figures 3G–I**). Moreover, the 1-year AUCs of the risk model in the TCGA testing cohort, the TCGA-COAD cohort and the GEO cohort were 0.707, 0.821 and 0.655, respectively, validating the good predictive performance of this model in patients with COAD (**Figures 3J–L**).

### Relationships between the ICRG prognostic signature and clinical characteristics

**Figure 4:**
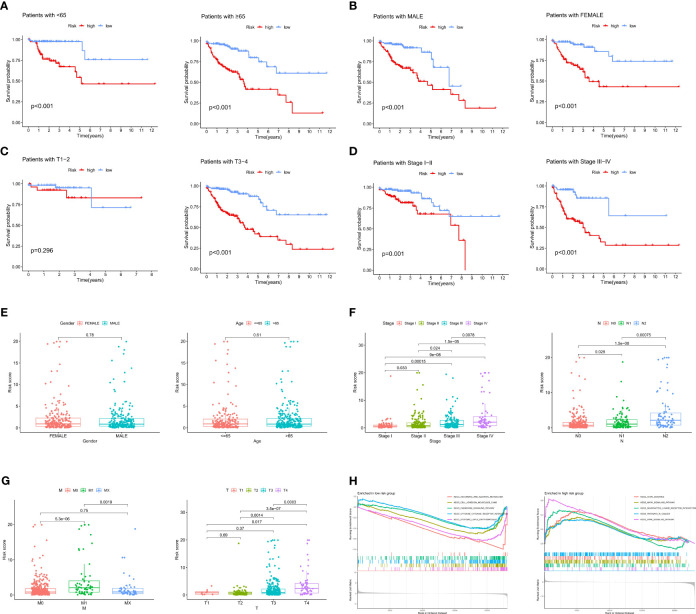
Correlations between the ICRG prognostic signature and clinical features in patients with COAD. **(A–D)** Kaplan-Meier analysis of overall survival (OS) in COAD patients assorted by **(A)** age (<65 vs. ≥ 65 years), **(B)** sex (males vs. females), **(C)** TNM stage (TI-II vs. TIII-IV) and **(D)** tumor stage (I-II vs. III-IV). **(E–G)** Relationships between risk scores (RS) and clinical characteristics, including age, sex, TNM stage and tumor stage. **(H)** Results of GSEA enrichment analysis in both the high-risk and low-risk groups.

To further explore the associations between the ICRG prognostic signature and patients’ clinical characteristics, RSs were compared in the TCGA-COAD cohort using independent t tests. Based on their clinical characteristics, patients were grouped into high and low risk groups and differences in prognosis were determined. Prognosis was significantly worse in patients in the high-risk than in the low-risk group based on clinical characteristics, such as age (P<0.001), gender (P<0.001), T3-4 status (P<0.001) and stage (P<0.001) (**Figures 4A–D**). Analyses of differences in RSs between groups classified by clinical features showed that RS was not affected by age or gender (**Figure 4E**). In contrast, RSs increased gradually and significantly as tumor stage and TNM increased (**Figures 4F, G**). These results demonstrated that this prognostic signature based on ICRGs showed a high degree of overall predictive power across various clinical characteristics.

In addition, the entire TCGA-COAD cohort was subjected to gene set enrichment analysis (GSEA) to identify gene sets significantly associated with both the low-risk and high-risk groups. Genes enriched in the low-risk group were associated with chemokine and cytokine pathways, whereas genes enriched in the high-risk group were associated with tumor-related signaling pathways (**Figure 4H**). Pathway enrichment analysis therefore showed that changes in signaling pathways and chemokines could lead to differences in immune states in low- and high-risk groups.

### Comparison of the ICRG prognostic forecasting model with other published models

**Figure 5:**
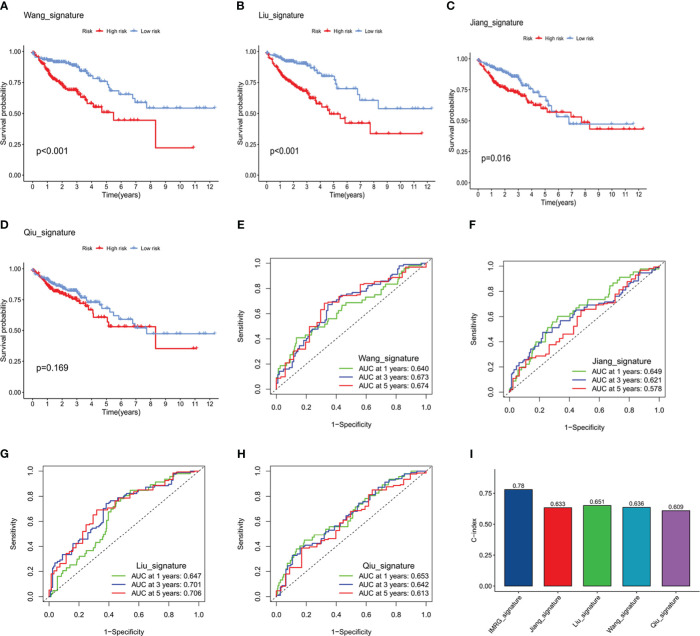
Comparison of the predictive accuracy of the ICRG prognostic signature with that of four previously published signatures. **(A-D)** Kaplan–Meier survival curve analysis of the four published signatures. **(E-H)** ROC curves showing the predictive accuracy of the four published signatures. **(I)** Comparison of the C-indices of the ICRG prognostic model with that of the other four prognostic models.

The relative predictive ability of the immune- and CAF-associated model described in this study was compared with the predictive ability of four previously-described prognostic models (12–15). To ensure the comparability of these signatures, the same method for calculating and converting the RS was applied to the entire TCGA-COAD cohort. Three of the previously published signatures were effective in categorizing the COAD samples into high- and low-risk groups, with the differences being statistically significant (**Figures 5A–D**). However, ROC curve analysis showed that the AUCs in the present model were higher than those of the four previously published signatures. Specifically, the present model had AUCs of 0.821, 0.803, and 0.732 for 1-, 3-, and 5-year survival, respectively (**Figures 5E–H**). In addition, the C-index of the present model was highest at 0.78, whereas the four other signatures had C-indices of 0.651 (16), 0.633 (17), 0.636 (18), and 0.609 (19). These results suggest that the prognostic performance of the ICRG prognostic signature consistently outperformed other evaluated signatures (**Figure 5I**).

### Development of a nomogram using the ICRG prognostic signature and assessment of its clinical relevance

**Figure 6:**
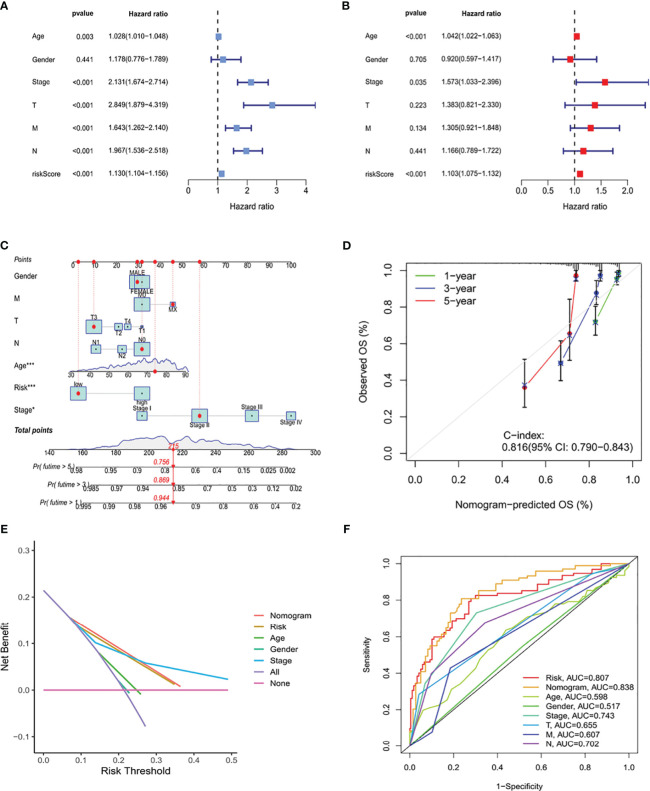
Construction of a nomogram based on the ICRG prognostic signature and evaluation of its clinical significance in the TCGA-COAD cohort. **(A, B)** Univariate and multivariate Cox regression analyses assessing the relationships between risk scores (RS) and clinical characteristics of patients in the TCGA-COAD cohort. **(C)** Development of a nomogram model predicting 1-, 3-, and 5-year overall survival (OS) in the TCGA-COAD cohort. The nomogram assigned points to each variable, with the points added to calculate a total score for each patient. Based on this score, the bottom scale was used to predict the probability of OS at the specified time points. **(D)** Calibration curve evaluating the agreement between the predicted probabilities of survival at 1-, 3-, and 5-years generated by the nomogram and the actual survival outcomes. The graph visually displays the degree of consistency between the predicted and observed survival rates. **(E)** DCA curve analysis of the clinical value of the nomogram model. **(F)** Comparison the ROC curves of clinical factors and the risk model. The nomogram model demonstrates better accuracy and performance in predicting the survival of patients with COAD.

The clinical suitability of the ICRG prognostic signature was determined by Cox regression analyses of the TCGA-COAD cohort. RS correlated significantly with prognosis on both univariate (P < 0.001) (**Figure 6A**) and multivariate (P < 0.001) (**Figure 6B**) regression analyses. A reliable nomogram predicting survival risk for individuals was constructed based on multiple regression analysis, which found that three variables, age, stage, and RS, had P values <0.05 (**Figure 6C**). Moreover, calibration curves suggested a strong correlation between the survival rates predicted by the nomogram and the actual survival rates (**Figure 6D**).

Decision curve analysis (DCA) can be used to evaluate the practical clinical benefit of the nomogram. These curves are based on a series of possible thresholds and can compare the net benefit of the model with other decision strategies. If the net benefit of the nomogram was higher than that of other decision strategies, then this model was considered to have clinical value. DCA showed that the nomogram had better predictive ability than any other predictors (**Figure 6E**). In additionally, the nomogram had an AUC of 0.838, outperforming other variables (**Figure 6F**). Thus, these findings showed that the ICRG-based nomogram correlated significantly with patient prognosis, suggesting that this nomogram could effective aid in predicting cancer progression.

### Ability of the ICRGs prognostic signature to predict response to immunotherapy

**Figure 7:**
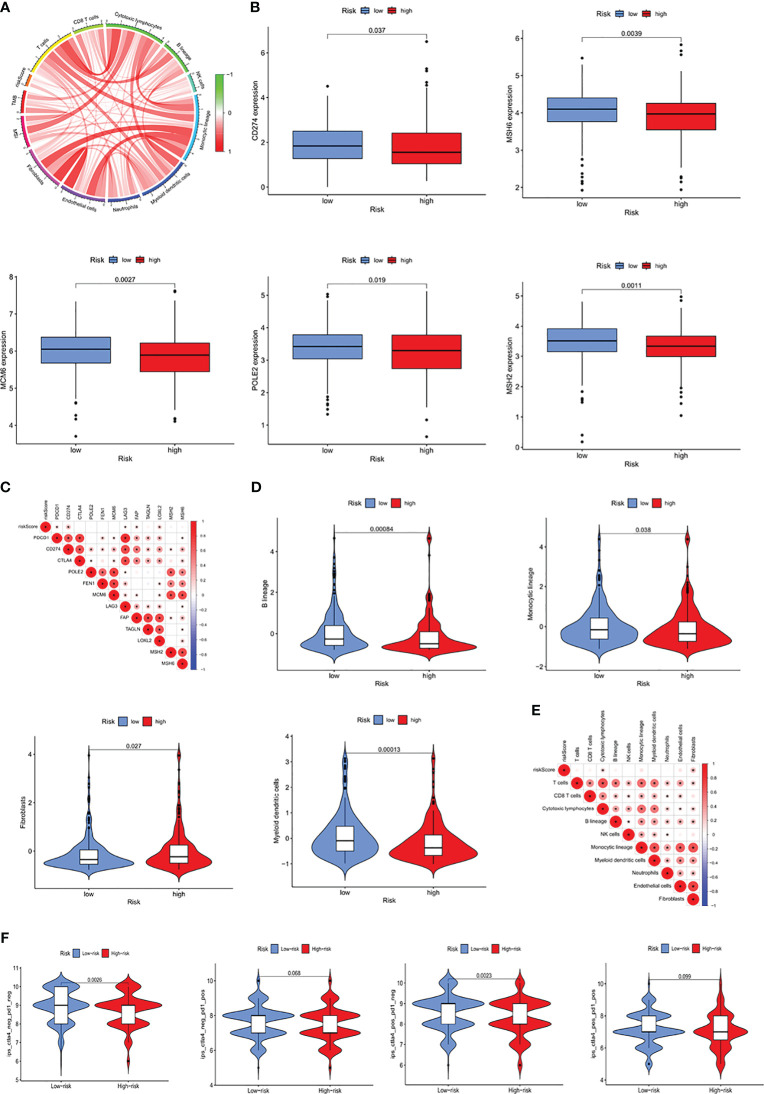
Prognostic ability of the ICRG signature to predict patient response to immunotherapy. **(A)** Correlation analyses of RS, MSI, and immune-related cells. **(B)** Comparative expression of immune checkpoint molecules (e.g. *CD274, MSH6, MCM6, POLE2* and *MSH2*) in the high- and low-risk groups. **(C)** Heatmap showing the correlations between RS and immune checkpoint expression. **(D)** Comparative immune cell infiltration in the high- and low-risk groups. **(E)** Heatmap showing the correlations between RS and immune cell infiltration. **(F)** Correlation between RS and four IPS scores associated with a single ICI (anti-CTLA4 or anti-PD1) or their combination.

To better understand the impact of the IMRG prognostic signature on immunotherapy outcomes, the correlations between RSs and the level of immune infiltration within the TME were analyzed. RS showed a positive correlation with the infiltration of cytotoxic lymphocytes and fibroblasts (**Figure 7A**). Moreover, the levels of expression of immune checkpoint proteins, including *CD274, CTLA4, MSH6, MCM6, POLE2*, and *MSH2*, were found to differ significantly in the high- and low-risk groups (**Figure 7B**), indicating a close relationship between RS and immune checkpoint proteins (**Figure 7C**). The proportions of B cells, monocytes, and myeloid dendritic cells were lower, whereas the proportions of fibroblasts were higher, in the high- than in the low-risk group (**Figure 7D**). Furthermore, correlation analysis showed that RS correlated significantly with cytotoxic lymphocytes and fibroblasts (**Figure 7E**). Analysis of the correlation between RS and IPS, which are valuable predictors of the effectiveness of immunotherapy, showed significant differences in IPS and IPS-CTLA4 between the high- and low-risk groups (**Figure 7F**). These results suggested that the prognostic signature based on ICRGs could indicate immune infiltration status and predict patient response to immunotherapy.

### Downregulation of S1PR5 improved the efficacy of anti-PD1 treatment in CRC

**Figure 8:**
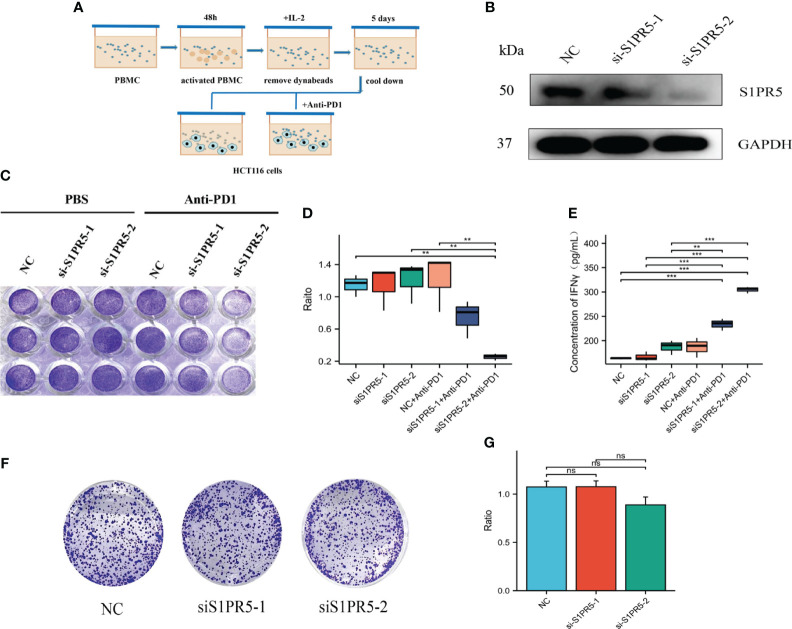
Effect of S1PR5 knockdown on the therapeutic efficacy of anti-PD1 monoclonal antibody. **(A)** Diagram of the T-cell killing assay. **(B)** Effect of si-S1PR5 on the expression of S1PR5 protein, as shown by western blotting. **(C-E)** Effect of S1PR5 knockdown and anti-PD1 antibody on T-cell cytotoxicity, as shown by crystal violet staining, with fewer tumor cells and a smaller staining area indicating stronger T-cell killing ability. Knockdown of S1PR5 alone did not significantly enhance T-cell killing ability (p>0.05), whereas the combination of S1PR5 knockdown and treatment with anti-PD1 antibody significantly enhancing T-cell killing ability (p<0.05). The expression level of IFNγ in cell culture supernatants is detected using ELISA method. Knocking down S1PR5 alone or using PD1 monoclonal antibody treatment did not significantly upregulate the expression levels of IFNγ, while the combination of s1PR5 knockdown with PD1 monoclonal antibody treatment significantly upregulated the expression levels of IFNγ (P<0.001). **(F, G)** Clonogenic assay, showing that S1PR5 knockdown did not affect the proliferation of HCT116 cells (p>0.05).

T cell killing experiments were performed to verify the role of S1PR5 in CRC immunity. PBMCs from healthy donors were activated for 48 hours with CD3/CD28 beads to obtain aPBMCs. To reduce non-specific killing, aPBMCs were incubated in the cold for 5 days and co-cultured with HCT116 cells in which S1PR5 had been knocked down, followed by the addition of anti-PD1 to test the effect of S1PR5 on T cell killing ability (**Figure 8A**). Western blotting showed that transfection of S1PR5 siRNA downregulated S1PR5 protein expression in HCT116 cells (**Figure 8B**). Crystal violet staining results showed that knock down of S1PR5 did not significantly increase the cytotoxic capacity of T cells, whereas the addition of anti-PD1 monoclonal antibody significantly enhanced the cytotoxic capacity of T cells (P<0.01) (**Figures 8C, D**). IFNγ was one of the markers of T cell activation and can effectively reflect the cytotoxicity of T cells. We concurrently performed an ELISA-based detection of the protein expression level of IFNγ in the cell supernatant. The experimental results indicated a significant upregulation in the expression of IFNγ in the group with knockdown of S1PR5 combined with PD1 monoclonal antibody (P<0.001), suggesting that the knockdown of S1PR5 significantly enhanced the efficacy of PD1 monoclonal antibody, thereby promoting the cytotoxicity of T cells (**Figure 8E**). To exclude cytotoxicity resulting from cell proliferation, the effects of S1PR5 on colorectal cancer cell proliferation were evaluated by testing clone formation. S1PR5 knockdown did not affect clone formation by HCT116 cells, suggesting that S1PR5 does not affect tumor proliferation (P>0.05) (**Figures 8F, G**). Taken together, these results indicate that knocking down S1PR5 can effectively enhance the therapeutic efficacy of anti-PD1 and promote the killing ability of T cells, suggesting that inhibition of S1PR5 could promote the therapeutic effects of anti-PD1.

## Discussion

The emergence of cancer immunotherapies and immune checkpoint inhibitors (ICIs) has enhanced the ability to treat cancer patients. To date, the programmed cell death-1 (PD-1)/programmed cell death ligand-1 (PD-L1) signaling pathway has been the most extensively studied pathway in tumor immunotherapy (20). Activation of this pathway can inhibit T cell proliferation, differentiation and secretion of cytokines, thus inhibiting T cell activity, impairing tumor immunosurveillance and triggering tumor immune tolerance and escape (21). Although ICIs have changed the treatment pattern of many tumors, the therapeutic effects of ICIs in some tumors are not obvious. One of the main factors affecting the therapeutic effects of ICIs is the complex TMEs, which are composed of CAFs and immune cells. CAFs play an important role in tumor immunity. Activation of the immune system and T cells can trigger the expression of multiple inflammatory cytokines by CAFs (22). This can result in a polarized imbalance of immune cells in the TME, making it difficult even for existing immune cells to effectively attack tumor cells (22). Simultaneously, CAFs can inhibit the function of immune cells, reducing the effectiveness of immune responses (23). The significant roles played by immune cells and CAFs in the TME suggest that the model described in the present study, based on the expression of immune and CAF-related genes, will accurately predict prognosis in patients with COAD. A thorough evaluation of immune and CAF-related genes in COAD can aid in the identification of new methods and pathways that can improve the efficacy of immunotherapy and enhance patient prognosis.

Co-cultivation of aPBMCs with CAFs enabled detection of changes in gene expression levels in CAFs and identification of CAF-related genes. Combining CAF-related genes with immune genes enabled identification of ICRGs, including those differentially expressed in the TCGA database. The TCGA-COAD cohort was divided into three subtypes using the NMF algorithm, and 1095 DEGs were classified. Findings from the ESTIMATE (24) and MCP counter showed that the degree of immune cell infiltration was higher in Cluster 2 than in Clusters 1 and 3, a difference that may have contributed to poorer prognosis in Cluster 2. These findings also suggest that the TME in Cluster 2 may be immunosuppressive. A prognostic signature based on 12 ICRGs was assessed in the TCGA training cohort using univariate and LASSO Cox regression analyses. The resulting predictive model categorized patients into high and low-risk groups based on their median RS, with further analysis showing that pathological and TNM stages were more advanced in the high-risk group. Regardless of clinical factors, however, this prognostic model showed exceptional predictive performance and was successfully validated in both internal and external cohorts. The C-index of this ICRG prognostic signature was notably better than the C-indices of four previously described signatures. Overall, these findings indicate that the prognostic signature based on ICRGs has superior prognostic ability than other prognostic signatures.

The ICRG model described in this study was based on 12 genes, all of which are involved in both tumors and the immune system. For example, CD1B plays significant roles in antigen presentation in the immune system (25) and in the progression of various solid malignancies (18). The present study showed that the gene with the highest coefficient was S1PR5 (0.90782435), suggesting that higher levels of expression of S1PR5 in CRC patients were associated with greater risk of progression and poorer prognosis. Sphingosine-1-phosphate (S1P), a metabolite of cell membrane sphingolipids, is a ubiquitous lysophospholipid signaling molecule that regulates various biological functions through binding to five subtypes of S1P receptors (S1PR1–S1PR5), all of which belong to the family of G-protein coupled receptors (GPCRs). Inhibitors have been developed against all S1PRs or specific S1PRs, with some of them being utilized clinically as immunomodulators. For example, fingolimod is an inhibitor that binds to S1PR1, 3, 4, and 5 (16).

Although S1PR5 was originally believed to be primarily located in the nervous system, recent research has indicated that it is also involved in the proliferation and migration of gastric and esophageal cancer cell lines (17). For example, the level of expression of S1PR5 was found to be significantly higher in malignant than in benign colon tissues (19). However, the role of S1PR5 in CRC immunity has not yet been determined. The results of the present study suggested that knocking down S1PR5 can significantly promote T cell killing ability and enhance the therapeutic effect of anti-PD1 antibody. These results indicated that S1PR5 played an important role in the development of CRC, and may become a new target in the treatment of CRC.

The present study had several strengths. First, the prognostic ICRG signature was validated in several datasets, including internal and external cohorts, making it highly reliable and robust. Second, a highly useful nomogram was developed to assist in quantitative calculations, suggesting that this nomogram may be useful in clinical applications. Third, this study found that S1PR5 could affect T-cell cytotoxicity, making it a potential target for intervention.

This study also had several limitations. Most importantly, the development of both the ICRG prognostic signature and the nomogram was based on a retrospective analysis of data. This prognostic signature and nomogram will therefore require validation in large multicenter prospective patient cohorts.

## Conclusion

The present study described the development of an ICRG prognostic signature, which incorporated immune- and CAF-related genes. This signature was found to be more accurate in predicting both prognostic risk and the efficacy of immunotherapy in patients with COAD. This prognostic signature was subsequently used to develop a personalized quantitative nomogram, which can be valuable in designing personalized treatments of patients with COAD.

## Data availability statement

The datasets presented in this study can be found in online repositories. The names of the repository/repositories and accession number(s) can be found in the article/**Supplementary Material**.

## Ethics statement

The studies involving humans were approved by Human Body Research Ethics Committee of the Second Hospital Affiliated to Zhejiang University School of Medicine. The studies were conducted in accordance with the local legislation and institutional requirements. The participants provided their written informed consent to participate in this study.

## Author contributions

JW: Writing – original draft. XG: Writing – original draft. YQ: Writing – original draft. KJ: Writing – review & editing. XC: Writing – review & editing. WL: Writing – review & editing. HY: Writing – review & editing. DF: Writing – review & editing. YF: Writing – review & editing. YZ: Writing – review & editing. QX: Writing – review & editing. YT: Writing – review & editing. KD: Writing – review & editing.
