# Supplementary figures and images for: Development and verification of a combined immune- and cancer-associated fibroblast related prognostic signature for colon adenocarcinoma
Source: Front Immunol. 2024 Jan 19;15:1291938. doi: 10.3389/fimmu.2024.1291938 (PMC10834644; doi:10.3389/fimmu.2024.1291938)

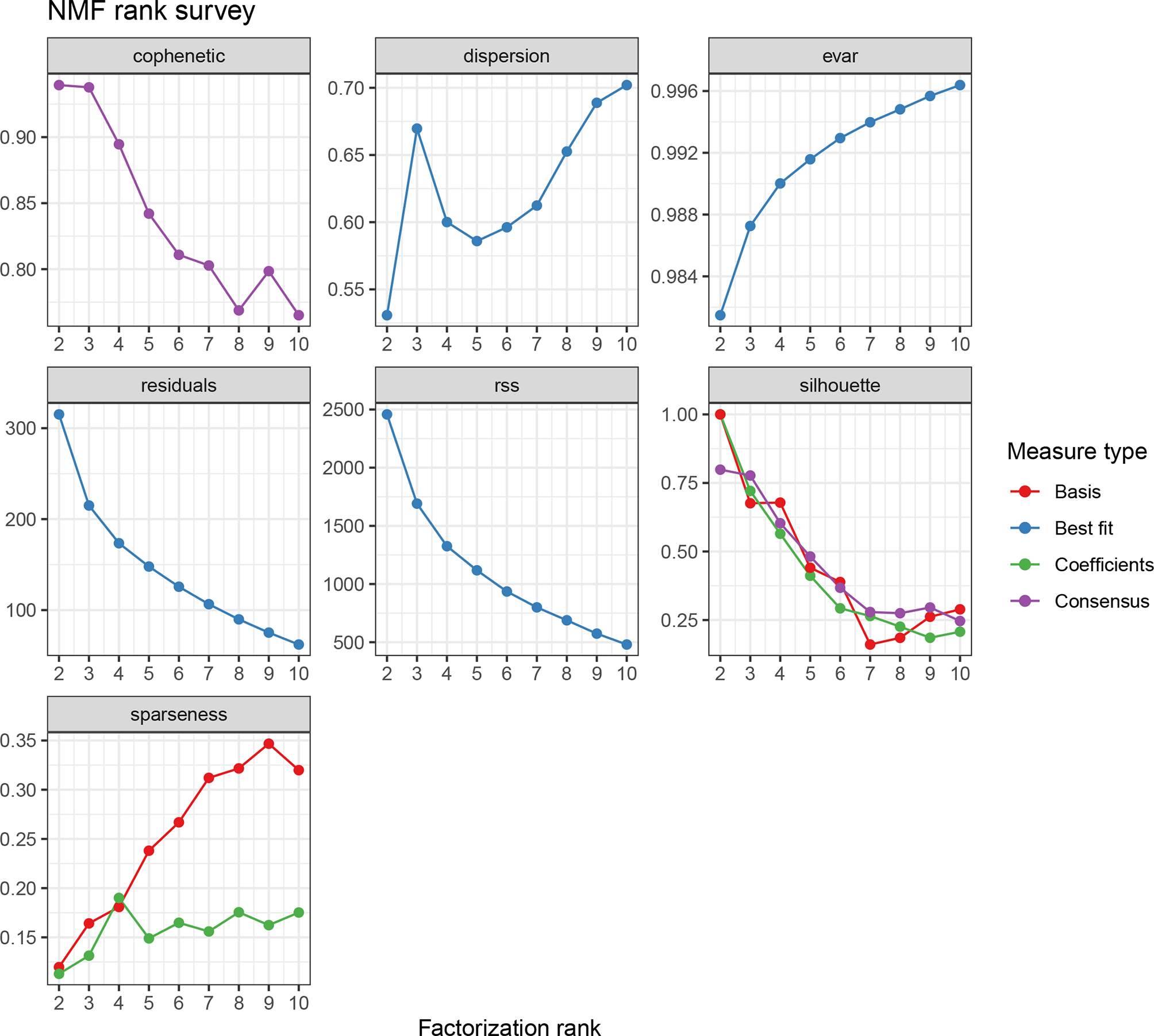

Supplement: Supplementary file 1 [file Image_1.tif]

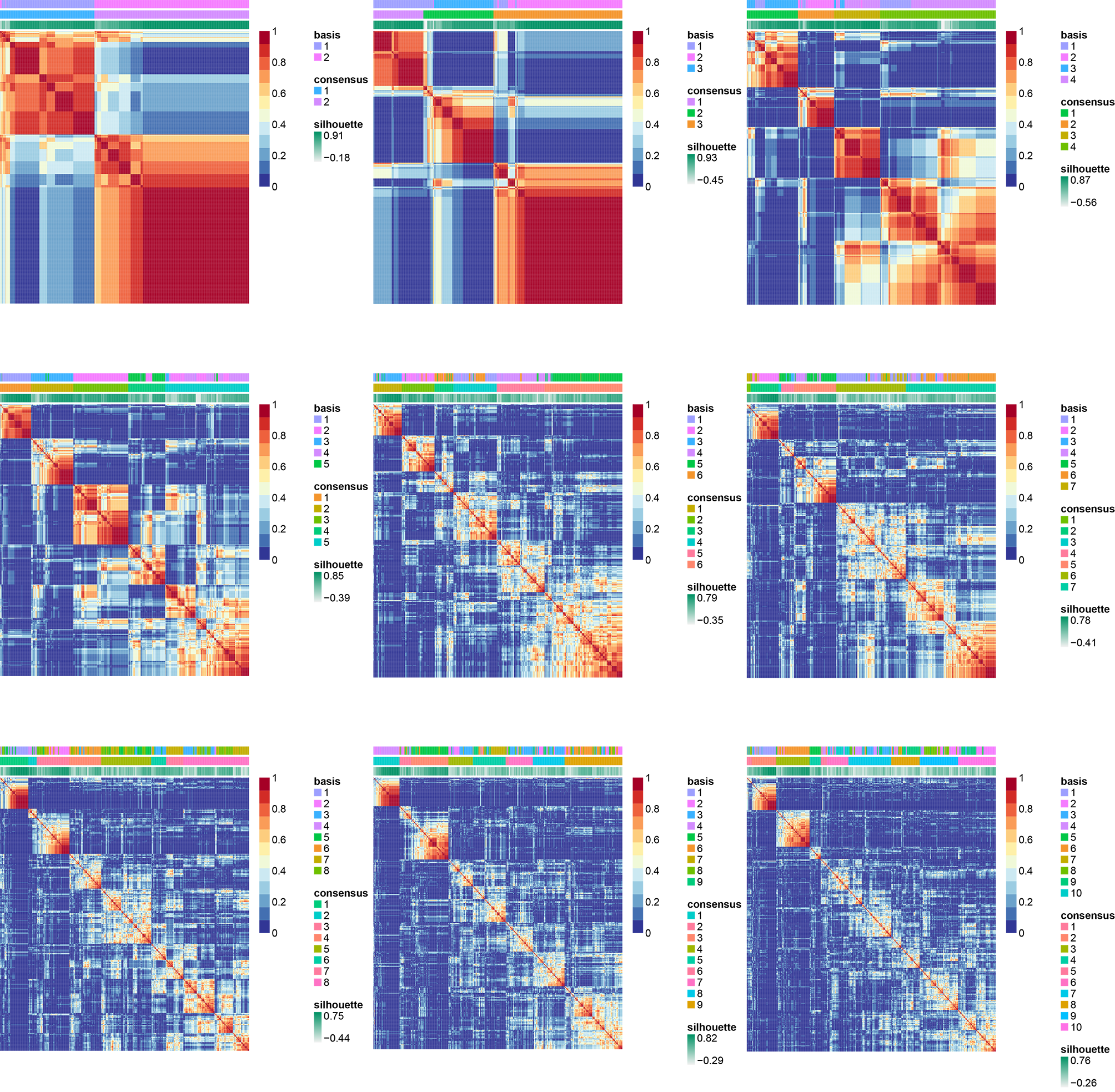

Supplement: Supplementary file 2 [file Image_2.tif]
